# The Chemokine CCL4 Stimulates Angiopoietin-2 Expression and Angiogenesis via the MEK/ERK/STAT3 Pathway in Oral Squamous Cell Carcinoma

**DOI:** 10.3390/biomedicines10071612

**Published:** 2022-07-06

**Authors:** Chien-Chi Lu, Hsiao-Chi Tsai, Dong-Ying Yang, Shih-Wei Wang, Ming-Hsui Tsai, Chun-Hung Hua, Kwei-Jing Chen, Michael Yuan-Chien Chen, Ming-Yu Lien, Chih-Hsin Tang

**Affiliations:** 1 Department of Otorhinolaryngology, China Medical University Hospital, Taichung 404327, Taiwan; Lucas12102012@gmail.com (C.-C.L.); minghsui@mail.cmuh.org.tw (M.-H.T.); eric-hua@hotmail.com (C.-H.H.); 2 School of Medicine, China Medical University, Taichung 404328, Taiwan; moxa0110@gmail.com; 3 Division of Hematology and Oncology, Department of Internal Medicine, China Medical University Hospital, Taichung 404327, Taiwan; 4 Graduate Institute of Basic Medical Science, China Medical University, Taichung 404328, Taiwan; fe2o33co2fe3co2@hotmail.com.tw; 5 Institute of Biomedical Science, Mackay Medical College, New Taipei City 252005, Taiwan; shihwei@mmc.edu.tw; 6 Department of Medicine, Mackay Medical College, New Taipei City 252005, Taiwan; 7 School of Pharmacy, College of Pharmacy, Kaohsiung Medical University, Kaohsiung 807378, Taiwan; 8 School of Dentistry, China Medical University, Taichung 404328, Taiwan; speach1026@gmail.com (K.-J.C.); mychen@mail.cmuh.org.tw (M.Y.-C.C.); 9 Department of Dentistry, China Medical University Hospital, Taichung 404327, Taiwan; 10 Graduate Institute of Biomedical Sciences, China Medical University, Taichung 404328, Taiwan; 11 Chinese Medicine Research Center, China Medical University, Taichung 404333, Taiwan; 12 Department of Biotechnology, College of Health Science, Asia University, Taichung 413305, Taiwan

**Keywords:** CCL4, angiopoietin 2, angiogenesis, oral squamous cell carcinoma, metastasis

## Abstract

Oral squamous cell carcinoma (OSCC) is a common malignant tumor with a poor prognosis and is a major public health burden in Taiwan. Angiogenesis, the formation of new blood vessels, promotes tumor proliferation, maintenance, and metastasis. Angiopoietin 2 (Angpt2), a mitogen with a strong angiogenic effect, is highly specific to endothelial cells and a key player in angiogenesis. The inflammatory chemokine (C-C motif) ligand 4 (CCL4) is also important in the pathogenesis and progression of cancer. In this study, an analysis of records from The Cancer Genome Atlas (TCGA) database found higher CCL4 expression in oral cancer tissue than in normal healthy tissue. CCL4 treatment of oral cancer cells upregulated Angpt2 expression and stimulated mitogen-activated protein kinase kinase (MEK), extracellular signal-regulated kinase 1/2 (ERK), and signal transducer and activator of transcription 3 (STAT3) phosphorylation. Transfection of oral cancer cells with MEK, ERK, and STAT3 inhibitors and their small interfering RNAs inhibited CCL4-induced promotion of Angpt2 expression and angiogenesis. In a mouse model of OSCC, CCL4-treated cells promoted neovascularization in implanted Matrigel plugs, whereas inhibiting CCL4 expression suppressed Angpt2 expression and angiogenesis. CCL4 shows promise as a new molecular therapeutic target for inhibiting angiogenesis and metastasis in OSCC.

## 1. Introduction

Over 90% of oral cancer malignancies are squamous cell carcinomas (OSCCs) [1], making this type of cancer a major public health problem worldwide. In 2018, a total of 354,864 new cases of oral cancer and 177,384 deaths due to oral cancer were recorded worldwide [2]. In that same year, oral cavity, oropharynx and hypopharynx cancers were the fifth most common cancers in Taiwan’s general population, with an age-standardized incidence of 22.5 per 100,000 people [3]. In particular, these were the fourth most common cancer diagnoses amongst Taiwanese males in 2018, with an age-standardized incidence rate of 42.2 per 100,000 people [3]. Tobacco smoking, alcohol consumption and betel nut chewing are major risk factors for oral cancer [4]. Standard treatment for OSCC is surgery followed by radiation or chemotherapy [2], but are associated with poor survival (with overall 5-year survival rates of around 40–50%), despite improvements in treatment methods combining different chemotherapeutic agents and radiotherapy protocols over the past few decades [5]. Therapeutic outcomes and survival are compromised not only by nodal and distal metastases [6,7], but also relatively low responsiveness to treatment, treatment resistance, disease recurrence, and the fact that ~40% of patients with OSCC are diagnosed with advanced stage IV disease, which has a poor prognosis [4,8]. Angiogenesis drives cancer progression and metastasis [9] and promotes the formation of lymphatic vessels, resulting in lymphangiogenesis [10].

Among the regulators of angiogenesis, the angiopoietin family growth factor angiopoietin 2 (Angpt2) has been considered to have a central role [11]. Angpt2 expression is high in early-stage angiogenesis and low in quiescent mature vessels [11]. The significance of Angpt2 in metastasis is reflected by evidence showing that the inhibition of Angpt2 significantly disrupts a prevalent vascular pattern in hepatocellular carcinoma, i.e., Angpt2 is essential for the formation of vessels that encapsulate tumor clusters (VETC) and facilitate venous invasion of tumor cells; inhibiting VETC formation effectively suppresses VETC-dependent intrahepatic metastasis [12]. Moreover, in adenocarcinoma, Angpt2 protein levels are significantly associated with advanced disease stage, poor survival, larger tumors and lymph node metastasis [13]. Much evidence points to the involvement of Angpt2 in the pathogenesis of various cancers, including OSCC, where evidence suggests that Angpt2 overexpression stimulates angiogenesis and increases metastasis in a mouse model of OSCC, potentially in part by inducing abnormal epithelial-mesenchymal transition (EMT)-induced angiogenesis [14].

The inflammatory chemokine (C-C motif) ligand 4 (CCL4), also known as macrophage inflammatory protein (MIP)-1β, has diverse effects on cytotoxic T lymphocytes and various types of immune and nonimmune cells [15,16]. CCL4 reportedly promotes tumor development and progression by recruiting tumor-associated macrophages and regulatory T cells [15,17]. The existing literature largely focuses on EMT and lymphangiogenesis as drivers of OSCC metastasis [18,19,20]; scant evidence has examined the contribution made by angiogenesis and especially by Angpt2, in OSCC lymph node metastasis [14]. For instance, CCL4-induced increases in vascular endothelial growth factor (VEGF) expression promote the proliferation, invasion and migration of tumor cells in endometrial cancer and OSCC [21,22]. We therefore sought to determine the relationship between CCL4 and angiogenesis in OSCC. In particular, since the evidence is unclear regarding any connection between CCL4 and Angpt2, the study explored this aspect in OSCC.

This study aimed to explore the cellular and molecular mechanisms of CCL4 in OSCC. We demonstrate that CCL4 promotes Angpt2 expression in OSCC by activating signal transducer and activator of transcription 3 (STAT3) and mitogen-activated protein kinase kinase (MEK), signal-regulated kinase 1/2 (ERK) signaling, which subsequently enhances Angpt2-induced tumor angiogenesis. These findings suggest new opportunities for the development of OSCC treatments.

## 2. Materials and Methods

### 2.1. Materials

Anti-mouse monoclonal antibodies specific for MEK (sc-6252), p-ERK (sc-7383), ERK (sc-1647), p-STAT3 (sc-56747), CCL4 (sc-130330), and anti-rabbit monoclonal antibodies specific for STAT3 (sc-482) were purchased from Santa Cruz Biotechnology (Shanghai) Co., Ltd. (Pudong New District, Shanghai, China). CD31 (ab9498) and Angpt2 (ab153934) antibodies were purchased from Abcam (Cambridge, UK). P-MEK (2338s) was purchased from Cell Signaling Technology, Inc. (Taigen Bioscience Corporation, Taipei, Taiwan). Beta-actin antibody (GT5512) was purchased from GeneTex, Inc. (Irvine, CA, USA). The control, MEK, ERK and STAT3 small interfering RNAs (siRNAs) were purchased from GE Healthcare Dharmacon, Inc. (Lafayette, CO, USA). Invitrogen™ Lipofectamine™ 2000 was purchased from Thermo Fisher Scientific (Carlsbad, CA, USA). Human CCL4 recombinant protein was purchased from PeproTech US (Cranbury, NJ, USA). Matrigel was purchased from Corning Life Sciences (Tewksbury, MA, USA). All other chemicals were purchased from Merck SA (Darmstadt, Germany).

### 2.2. Cell Culture

The human tongue squamous carcinoma cell lines used in this study were SAS and SCC4. The SAS cell line was purchased from the Japanese Collection of Research Bioresources Cell Bank (JCRB, Shinjuku, Japan) (JCRB No: JCRB0260) and the SCC4 cell line was purchased from the Bioresource Collection and Research Center (BCRB, Hsinchu, Taiwan) (BCRC No: 60142). Cells were cultured at 37 °C in a humidified atmosphere of 5% CO_2_ and in Dulbecco’s Modified Eagle Medium with 10% fetal bovine serum.

### 2.3. The Cancer Genome Atlas (TCGA) Data Preparation

Transcriptomic profiling data of OSCC mucosal tissues (the tongue, alveolar ridge, oral cavity, hard palate, the mouth floor, buccal mucosa) held by the TCGA database were downloaded using the Broad Institute GDAC Firehose [23] (http://gdac.broadinstitute.org). A total of 350 OSCC samples and 44 normal oral mucosal tissues were analyzed for gene expression profiles of *CCL4* and *ANGPT2*.

### 2.4. Collection of Tissue Specimens from OSCC Patients

Sixteen patients newly diagnosed with OSCC scheduled to undergo surgery in China Medical University Hospital (Taichung, Taiwan) were recruited for this study. Clinicopathological features were collected from medical records. All patients were staged according to the 8th edition of the American Joint Committee on Cancer’s (AJCC) Cancer Staging Manual [24]. The study was granted research access to surgically excised OSCC specimens matched with non-tumor epithelial tissues collected from each patient. The study was conducted according to the guidelines of the Declaration of Helsinki, and approved by the Institutional Review Board of China Medical University Hospital. All patients gave written consent before enrollment.

### 2.5. Quantitative Real-Time PCR

Quantitative real-time PCR was performed using the StepOnePlus™ sequence detection system, according to a previous protocol (22). The following *ANGPT2* primer sequences were used: forward 5′-GGCAGCGTTGATTTTCAGAGGACT-3′ and reverse 5′-TTTAATGCCGTTGAACTTATTTGT-3′. The level of *GAPDH* expression was used as the endogenous control for normalization of each sample.

### 2.6. CCL4 Knockdown and Lentiviral Infection

CCL4 knockdown was performed using the short hairpin RNA (shRNA) expression lentivirus system containing the specific shRNA (target sequence: GTGCTGATCCCAGTGAATCCT) in the pLKO.1-puro vector generated in 293T cells. The plasmid and negative control (control shRNA) were purchased from the National RNAi Core Facility of Academia Sinica (Taipei, Taiwan). For producing lentivirus, 293T cells were transfected using JetPRIME transfection reagent with 4 μg of shCCL4 pLKO.1 vectors, 1 μg of pVSV-G plasmid, and 3.6 μg of pCMVΔR8.91 plasmid. After 48 h of incubation, the virus solution was collected. OSCC cells were infected with the prepared lentivirus (preincubated with 8 µg/mL of polybrene) for 24 h. The medium was then changed to DMEM medium containing 10% fetal bovine serum and 4 µg/mL of puromycin.

### 2.7. In Vivo Matrigel Plug Assay

Four-week-old nude BALB/c mice were subcutaneously injected with 0.1 mL of liquid Matrigel (10.4 mg of protein/mL) mixed with 0.1 mL culture medium (CM) at a final concentration equal to 5.2 mg/mL. Before this injection, the mice were exposed to volatile anesthesia consisting of a total of 3% isoflurane mixed in 100% oxygen as carrier gas delivered in a closed chamber for 3–5 min. After loss of the righting reflex, the mice were transferred to a heating pad (36.5 °C) to minimize pain and distress during injection of the plugs. All mice were housed under a 12/12 h light/dark cycle and were checked daily for survival. On Day 14, all mice were euthanized by CO_2_ and the Matrigel plugs were excised and quantified for the extent of blood vessel formation, according to previously detailed procedures [25]. All animal experiments were conducted in accordance with a protocol approved by the Institutional Animal Care and Use Committee of China Medical University (CMUIACUC-2020-132).

### 2.8. Immunohistochemistry (IHC)

Before proceeding with the staining protocol, the tissue sections were deparaffinized by xylene and pretreated with graded ethanol (100%, 95%, 85%, 75%, 70%) for 5 min each in distilled water. Human CCL4, Angpt2 or CD31 antibody was applied at room temperature for 2 h. Data were scored using ImageJ software to quantify the percentages of positive detections for human CCL4, Angpt2 or CD31 and intensity of the staining.

### 2.9. Western Blot Analysis

After washing the cell culture dish with phosphate-buffered saline (PBS), cell lysates were prepared by ice-cold RIPA buffer containing a protease inhibitor cocktail. SDS-PAGE gels were used to separate protein samples, which were transferred to polyvinylidene difluoride (PVDF) membranes. The blots were blocked with 4% nonfat milk for 1 h. Next, after 3 washes in Tris-buffered saline with 0.05% Tween 20 (TBS-Tween), the blots were incubated with specific antibodies overnight at 4 °C. The bands were detected with HRP-conjugated secondary antibodies using the ECL chemiluminescent detection method.

### 2.10. Tube Formation Assay

Forty-eight-well plates were coated with Matrigel (100 μL/well) before use. Endothelial progenitor cells (EPCs) were seeded into each well in 0% MV2 medium and 10% CM at a 1:1 ratio, then incubated for 16 h at 37 °C. Quantitative data were obtained using ImageJ software by calculating tube branches and total tube lengths.

### 2.11. Cell Migration Assay

An in vitro cell migration assay was performed in a 24-well Transwell plate. EPCs were seeded onto the upper chamber and incubated in the lower chamber with 0% MV2 medium and 10% CM at a 1:1 ratio, then incubated for 24 h at 37 °C. Cell migratory ability was assayed according to the manufacturer’s instructions.

### 2.12. Statistical Analysis

All statistical data were analyzed using GraphPad Prism 8.0.2 (GraphPad Software, San Diego, CA, USA) and are expressed as the means ± standard deviations. Statistical comparisons between two samples were performed using the Student’s *t*-test; one-way analysis of variance (ANOVA) compared the means of more than two groups. A *p*-value of <0.05 was considered significant.

## 3. Results

### 3.1. Clinicopathological Characteristics of CCL4 and Angpt2 Expression in OSCC Tissue

**Figure 1:**
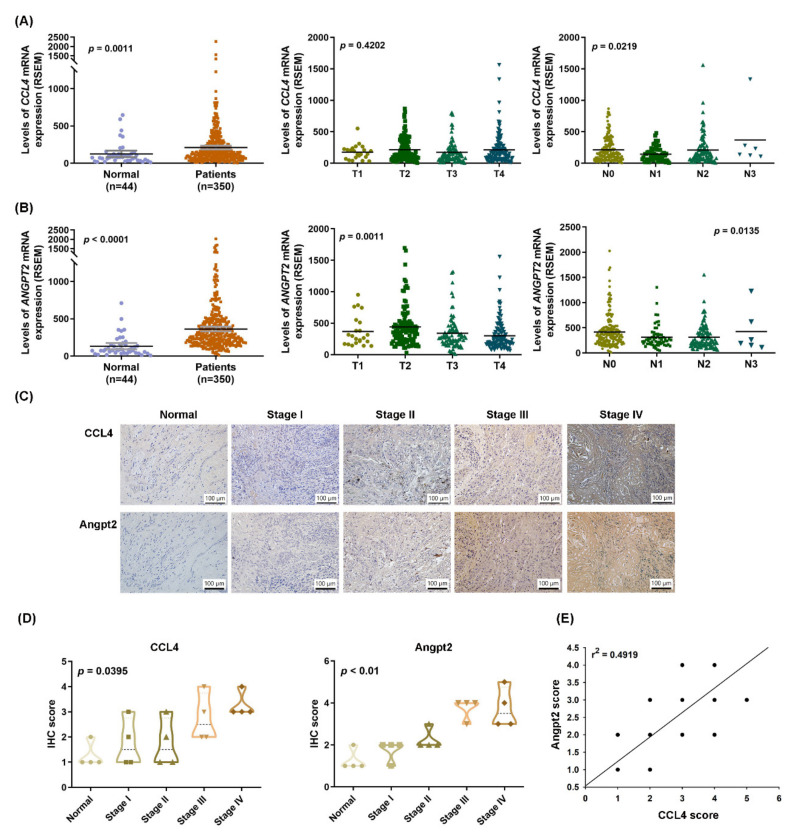
Clinicopathological characteristics of *CCL4* and *Angpt2* expression in OSCC tissue. (**A**,**B**) The expression profiles of *CCL4* and *ANGPT2* in tumor tissue and normal tissue specimens were analyzed in records downloaded from the TCGA database. (**C**,**D**) OSCC and adjacent normal tissue specimens from China Medical University Hospital were subjected to IHC evaluations with CCL4 or Angpt2 antibodies, and the positive staining percentages of the areas were scored as: 1 (<20%), 2 (20–40%), 3 (40–60%), 4 (60–80%), or 5 (80–100%). (**E**) A positive correlation was observed between CCL4 and Angpt2 expression (Spearman’s rank correlation coefficient, *r*^2^ = 0.4919).

This study used data from TCGA database to explore the gene profiles of *CCL4* and *ANGPT2* and their clinical significance. As shown in Figure 1A,B, we found higher levels of *CCL4* and *ANGPT2* expression in the 350 samples of OSCC tissue compared with 44 normal oral mucosal tissue samples. Univariate analysis revealed that higher pathological N status according to AJCC tumor-node-metastasis (TNM) classification was significantly correlated with higher *CCL4* and *ANGPT2* expression; only *ANGPT2* expression was positively correlated with pathological T status (Figure 1A,B). Protein levels of CCL4 and Angpt2 expression detected by IHC analyses were significantly higher in OSCC tissue than in adjacent normal tissue and were significantly associated with clinical disease stages (Figure 1C,D). In addition, we observed a significant correlation between levels of CCL4 and Angpt2 expression in tumor specimens (Figure 1E). These findings demonstrate that levels of CCL4 and Angpt2 are positively correlated with OSCC clinical disease stages.

### 3.2. CCL4 Increases Angpt2 Expression in OSCC Cells and Promotes Angiogenesis

**Figure 2:**
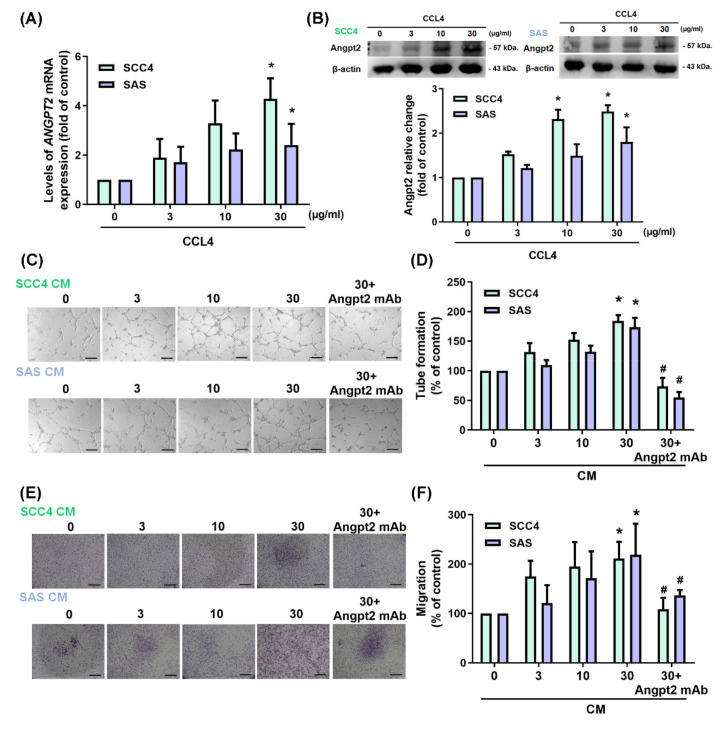
CCL4 increases Angpt2 expression and promotes angiogenesis in OSCC cells. (**A**,**B**) OSCC cells were treated with various concentrations of CCL4 protein (0–30 μg/mL), then levels of *ANGPT2* mRNA and protein expression were detected by qPCR and Western blot, respectively. (**C**,**D**) OSCC cells were treated with various concentrations of CCL4 (0–30 μg/mL) for 24 h. Culture medium was collected as CM and applied to EPCs for 16 h. The capillary-like structure formation was examined by the tube formation assay. Scale bar, 100 μm. (**E**,**F**) OSCC cells were treated with various concentrations of CCL4 (0–30 μg/mL) for 24 h. CM was applied to the EPCs for 24 h. EPC migration ability was examined by the Transwell migration assay. Scale bar, 50 μm. Each experiment was performed three times. * *p* < 0.05 compared with controls.; ^#^ *p* < 0.05 compared with the CCL4-treated group (30 μg/mL).

**Figure 3:**
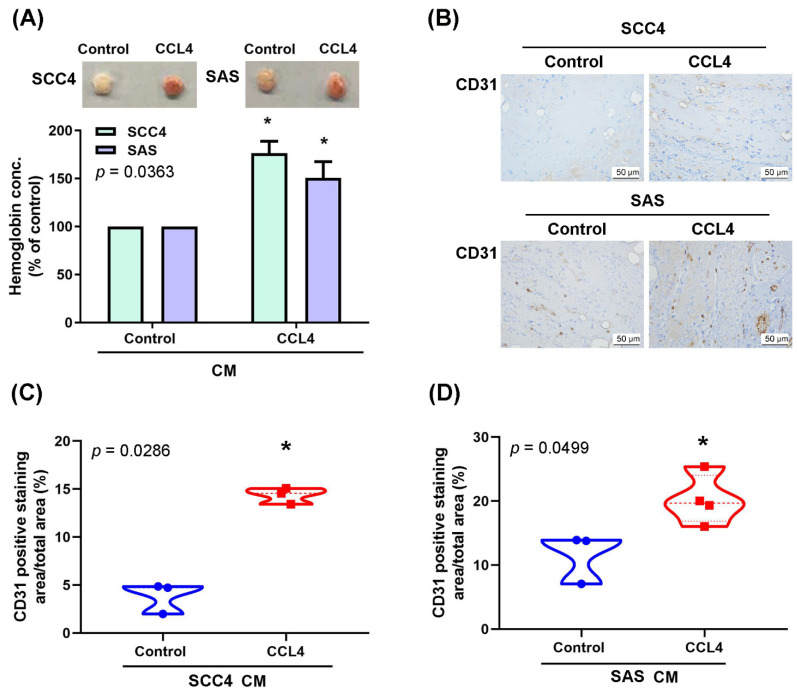
CCL4 promotes OSCC angiogenesis in vivo. Mice were injected subcutaneously with Matrigel containing 0.2 mL CM from OSCC cells (treated with or without CCL4). On Day 14, Matrigel plugs were excised, photographed, quantified for hemoglobin content (**A**), then stained with CD31 (**B**–**D**). * *p* < 0.05 compared with controls.

Treating the OSCC cell lines SCC4 and SAS with different concentrations of CCL4 dose-dependently upregulated levels of *ANGPT2* mRNA and protein expression (Figure 2A,B). Moreover, dramatic increases in EPC tube formation and migratory ability in CM from CCL4-treated cells were abolished by the Angpt2 monoclonal antibody (mAb) (Figure 2C–F). Results from the in vivo Matrigel plug assay revealed that Matrigel mixed with CM from CCL4-treated cells promoted neovascularization in the plugs, as assessed by hemoglobin concentrations (Figure 3A). The angiogenic response was also quantified by the sectioning of the Matrigel plug and assessing the levels of CD31 expression (well-defined markers of angiogenesis). As shown in Figure 3B–D, high levels of CD31-positive expression were found in the plugs of CM from CCL4-treated cells. These results indicate that CCL4 enhances OSCC angiogenesis by upregulating Angpt2 expression.

### 3.3. CCL4 Promotes Angpt2 Expression and Angiogenesis by Activating MEK and ERK Signaling

**Figure 4:**
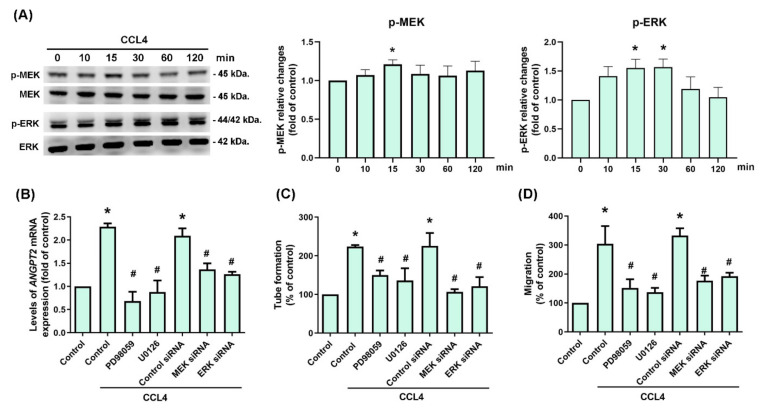
CCL4 promotes Angpt2 expression and angiogenesis by activating MEK and ERK signaling. (**A**) SCC4 cells were incubated with CCL4 (30 μg/mL) for the indicated time intervals, then MEK and ERK activation was examined by Western blot. (**B**) SCC4 cells were treated with a MEK inhibitor (PD98059, 10 μM) or ERK inhibitor (U0126, 10 μM) for 30 min, or transfected with MEK or ERK siRNAs for 24 h, then stimulated with CCL4 (30 μg/mL) for 24 h. Levels of *ANGPT2* mRNA were examined by qPCR. (**C**) SCC4 cells were treated with a MEK inhibitor (PD98059, 10 μM) or ERK inhibitor (U0126, 10 μM) for 30 min, or transfected with MEK or ERK siRNAs for 24 h, then stimulated with CCL4 (30 μg/mL) for 24 h. CM was applied to EPCs for 16 h. Capillary-like structure formation was examined by the tube formation assay. (**D**) SCC4 cells were treated with a MEK inhibitor (PD98059, 10 μM) or ERK inhibitor (U0126, 10 μM) for 30 min, or transfected with MEK or ERK siRNAs for 24 h, then stimulated with CCL4 (30 μg/mL) for 24 h. CM was applied to the EPCs for 24 h. EPC migratory ability was examined by the Transwell migration assay. Each experiment was performed three times. * *p* < 0.05 compared with controls; ^#^ *p* < 0.05 compared with the CCL4-treated group (30 μg/mL).

The MEK and ERK signaling cascade is considered to be the most important of all mitogen-activated protein kinase (MAPK) signaling pathways, and plays a crucial role in tumor angiogenesis [26]. We therefore incubated OSCC cell lines with CCL4, to examine whether MEK and ERK signaling is involved in OSCC angiogenesis. The data showed that the phosphorylation levels of MEK and ERK were increased after 15 and 30 min, respectively (Figure 4A). Pretreating cells with MEK or ERK inhibitors and their respective siRNAs prevented CCL4-mediated upregulation of *ANGPT2* mRNA expression (Figure 4B). Similarly, the blockade of MEK or ERK signaling by its inhibitors and siRNAs suppressed EPC tube formation and migration (Figure 4C,D). Thus, CCL4 appears to promote Angpt2 expression and angiogenesis by activating the MEK and ERK signaling pathway in human OSCC cells.

### 3.4. STAT3 Activation Is Involved in CCL4-Induced Upregulation of Angpt2 Expression and Angiogenesis

**Figure 5:**
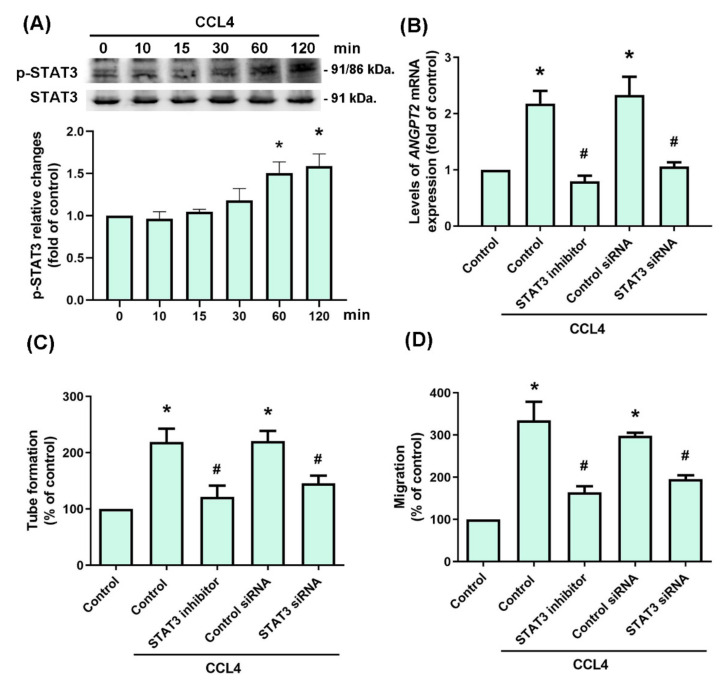
STAT3 activation was involved in CCL4-induced promotion of Angpt2 expression and angiogenesis. (**A**) SCC4 cells were incubated with CCL4 (30 μg/mL) for the indicated time intervals, before STAT3 activation was examined by Western blot. (**B**) SCC4 cells were treated with a STAT3 inhibitor (10 μM) or transfected with a STAT3 siRNA, then stimulated with CCL4 (30 μg/mL) for 24 h. Levels of *ANGPT2* mRNA were examined by qPCR. (**C**) SCC4 cells were treated with a STAT3 inhibitor (10 μM) or transfected with a STAT3 siRNA, then stimulated with CCL4 (30 μg/mL) for 24 h. CM was applied to EPCs for 16 h. Capillary-like structure formation was examined by the tube formation assay. (**D**) SCC4 cells were treated with a STAT3 inhibitor (10 μM) or transfected with a STAT3 siRNA, then stimulated with CCL4 (30 μg/mL) for 24 h. CM was applied to EPCs for 24 h. EPC migratory ability was examined by the Transwell migration assay. Each experiment was performed three times. * *p* < 0.05 compared with controls; ^#^ *p* < 0.05 compared with the CCL-treated group (30 μg/mL).

STAT3 phosphorylation and subsequent nuclear translocation is implicated in cellular invasion and angiogenesis in oral cancer [27,28]. In order to determine the mechanisms underlying CCL4-mediated angiogenesis of oral cancer, we treated OSCC cells with CCL4 for 120 min and observed time-dependent increases in STAT3 phosphorylation (Figure 5A). Inhibiting STAT3 expression significantly decreased levels of *ANGPT2* mRNA expression (Figure 5B), as well as EPC tube formation and migratory activity (Figure 5C,D). These results indicate that CCL4-induced Angpt2 expression and stimulation of angiogenesis occurs through STAT3 activation.

### 3.5. Silencing CCL4 Expression Inhibits Tumor-Induced Angiogenesis In Vivo

**Figure 6:**
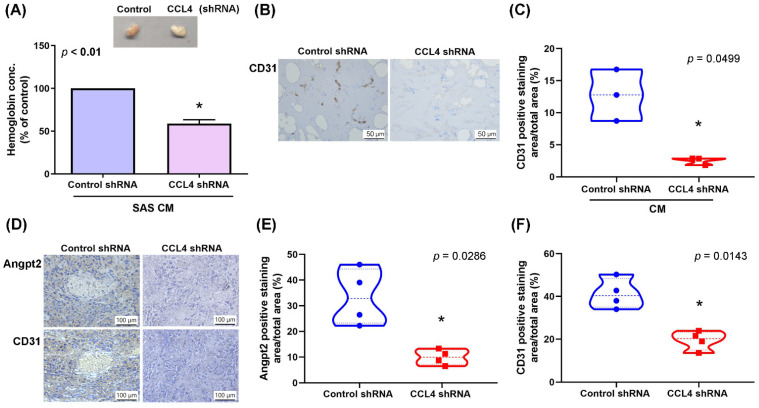
Silencing CCL4 expression inhibits tumor-induced angiogenesis in vivo. (**A**–**C**) Mice were injected subcutaneously with Matrigel OSCC CM for 14 days, after which time the plugs were excised, photographed and quantified for hemoglobin content (**A**), then stained with CD31 (**B**,**C**). (**D**–**F**) The tumor sections were immunostained using Angpt2 and CD31 antibodies. * *p* < 0.05 compared with controls.

We next investigated the role of CCL4 in vivo. CM collected from SAS cells stably expressing control or CCL4 shRNA was mixed with Matrigel and inoculated into nude BALB/c mice. On Day 14 after inoculation, neovascularization in plugs was abolished in the CCL4 shRNA group, as determined by hemoglobin concentrations (Figure 6A). IHC analysis also revealed that CCL4 knockdown reduced de novo neovessel formation, according to the results from CD31 staining (Figure 6B,C). We have previously confirmed that inhibiting CCL4 expression suppresses VEGF-C-induced lymphangiogenesis in vivo [22]. In this study, when we re-stained the tumor specimens with Angpt2 and CD31, we observed that levels of Angpt2 and CD31 expression in excised tumors were significantly decreased in the SAS/CCL4 shRNA cells (Figure 6D–F). These results suggest that silencing CCL4 inhibits Angpt2 expression and angiogenesis in vivo.

## 4. Discussion

Angiogenesis is essential for the development of OSCC [29]. Understanding the underlying mechanisms of OSCC angiogenesis may assist with the development of novel treatment approaches for OSCC, which has a low overall 5-year survival rate, despite improvements in surgical management and therapeutic combinations of radiation and chemotherapy [4]. In particular, late-stage OSCC has a very poor prognosis, especially due to the fact that as many as ~40% of OSCC patients fail to present for diagnosis until they have stage IV disease [8]. This situation is exacerbated by the fact that biomarkers are lacking for the identification of early-stage OSCC, its prognosis, and appropriate management [30]. Thus, clinicians need accurate indications of biochemical as well as immunological alterations in OSCC tumors, to decide on how best to treat their patients [30]. C-C motif chemokine ligands are crucial for the functioning of the tumor microenvironment [17]. Importantly, the ability of Spi-B, a lymphocyte lineage-specific Ets transcription factor, to promote the invasion of macrophages in human lung cancer is inhibited when the *CCL4* gene is deleted [31]. Not only are levels of CCL4 upregulated in human lung adenocarcinoma cancer tissue, but patients with higher CCL4 levels have shorter overall survival [32]. Furthermore, CCL4 promotes VEGF-C expression and lymphangiogenesis in OSCC cells; inhibiting CCL4 expression suppresses VEGF-C-induced lymphangiogenesis in OSCC cells [22]. In this study, we found that CCL4 greatly enhanced Angpt2 expression and promoted angiogenesis in OSCC cells, suggesting that higher levels of CCL4 can promote both lymphangiogenesis and angiogenesis in OSCC. Levels of CCL4 expression therefore appear to be appropriate biomarkers and therapeutic targets in OSCC, especially in OSCC metastasis.

Interestingly, while both Angpt2 and VEGF promote angiogenesis, these growth factors have distinct functions in tumor angiogenesis and metastasis [11]. Angpt2 functions on the stalk and the tip cell phenotypes of endothelial cells, the first step in what has been termed the “angiogenic switch” [33,34]; VEGF is involved in endothelial tube formation [35]. Studies on tumor angiogenesis and metastasis have been able to highlight the importance of Angpt2 in different cancers; for instance, Angpt2 overexpression promotes the invasive abilities of cervical cancer cells, while the blockade of Angpt2 significantly decreases the expression of vimentin (a marker for EMT) and microvessel density in mice bearing cervical tumors [36]. Similarly, the inhibition of Angpt2 markedly inhibits the progression of late-stage, metastatic mammary tumors and pancreatic insulinomas in murine tumor models [37]. It is also reported that compared with the individual inhibition of Angpt2 and VEGF, their co-inhibition reduces lesion permeability and brain metastases by greater extents in experimental breast cancer metastasis [38]. Similar synergistic effects have also been demonstrated by the simultaneous blockade of Angpt2 and VEGF in mice with endometrial cancer, where combined silencing more effectively reduced tumor size and angiogenesis than the individual targeting of either Angpt2 or VEGF [39]. Recent clinical trials have described promising outcomes from the use of therapies that block Angpt2 alone or in combination with other pathways, such as the VEGF signaling pathway [11,34]. In this study, Angpt2 mAb significantly inhibited CCL4-mediated angiogenesis. This highlights the potential efficiency of Angpt2 inhibitory strategies for cancer therapy.

STAT family members are both signal transducers and transcription factors [40]. Activation of STAT3 contributes to the development of many cancers [41], while the inhibition of STAT3 reportedly elicits apoptotic cell death and stimulates immune-related activities to eliminate cancer cells [41]. Previous research has demonstrated the importance of tumor cell-intrinsic and extrinsic STAT3 signaling, as these activities encourage angiogenesis in non-small cell lung cancer by upregulating the expression of VEGF and other growth factors [40]. In this study, our results indicated the involvement of MEK, ERK, and STAT3 phosphorylation in CCL4-mediated promotion of Angpt2 expression and angiogenesis. It appears that STAT3 protein expression is mediated via the ERK pathway [42], while ERK activation can modulate STAT3 signaling in oral cancer [43], although our study results did not clearly reveal whether STAT3 is directly or indirectly activated by ERK. Our results do suggest that STAT3-induced stimulation of Angpt2 expression is critical for human OSCC angiogenesis, indicating that STAT3 is an attractive target for the development of effective therapeutics that can combat OSCC.

Finally, it should be noted that several limitations exist in this study. Firstly, although our data strongly suggest that CCL4 promotes Angpt2-mediated angiogenesis in OSCC cells in vitro, we cannot exclude the possibility that CCL4 also promotes the activities of other proangiogenic factors, such as VEGF and basic fibroblast growth factor (bFGF). Secondly, in the in vivo Matrigel plug assay, CM from OSCC delivers proangiogenic factors locally within the Matrigel. Subsequent neovascularization occurs mainly via resident cells such as EPCs, endothelial cells, or macrophages, infiltrating into the Matrigel plugs. Thirdly, we acknowledge that species differences may exist when the Matrigel plug formation assay is used to study tumor cell behavior, as Matrigel is mouse-derived and from a basement membrane containing molecules such as laminin, type IV collagen, entactin and proteoglycans, which act as thin scaffolds that separate cells and support tissue structures [44]. Matrigel may not therefore be the ideal matrix for studying human OSCC cell behavior. Moreover, stiffness and viscosity of the tumor microenvironment (TME) affect the speed and distance of migrating cells; Matrigel-collagen is reportedly stiffer and more viscous than other TME matrices [45]. The recent introduction of the human-based extracellular matrix, Myogel, has provided cancer researchers the opportunity to work within a 3D setting that mimics TME matrices [45,46]. Another advantage of Myogel is that this matrix consists of several small molecular proteins, such as cytokines, growth factors and growth factor receptors, which promote human cancer cell invasion [46]. Myogel is therefore an ideal matrix for human cancer cell invasion assays, but our laboratory has not yet had the chance to experiment with Myogel. We hope to use Myogel to study human OSCC angiogenesis in future.

## 5. Conclusions

**Figure 7:**
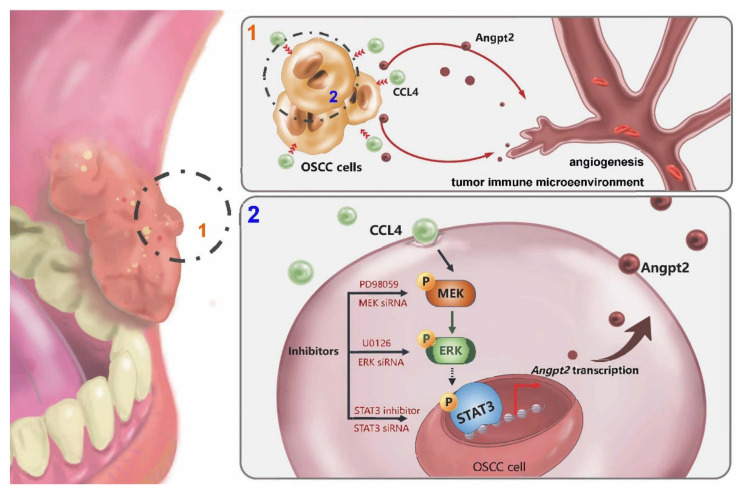
The schema depicts the involvement of signaling pathways in CCL4-induced stimulation of Angpt2 expression and the subsequent stimulation of OSCC angiogenesis.

In this study, data from the TCGA database demonstrated much higher gene levels of *CCL4* and *ANGPT2* expression in OSCC tissue samples compared with levels in normal tissue, and both CCL4 and Angpt2 expression correlated positively with OSCC clinical disease stages. Our cellular experiments indicated the involvement of MEK, ERK and STAT3 phosphorylation in CCL4-mediated promotion of Angpt2 expression and angiogenesis (Figure 7). Moreover, in a mouse model of OSCC, inhibiting CCL4 expression suppressed Angpt2 expression and angiogenesis. Thus, CCL4 may be a new molecular therapeutic target for inhibiting angiogenesis in OSCC.

## Data Availability

The data presented in this study are available on request from the corresponding author.
